# The complete mitochondrial genome of *Echinoecus nipponicus* Miyake, 1939 (Crustacea: Decapoda: Pilumnidae), a symbiont of sea urchins

**DOI:** 10.1080/23802359.2017.1419093

**Published:** 2017-12-21

**Authors:** Sang-Hui Lee, Taeg Kwan Oh, Myung-Hwa Shin

**Affiliations:** National Marine Biodiversity Institute of Korea, Seocheon, Republic of Korea

**Keywords:** *Echinoecus nipponicus*, symbiont crab, complete mitogenome, Eumedoninae, Decapoda

## Abstract

The mitochondrial genome of a crab symbiotic with sea urchins, *Echinoecus nipponicus*, was completely sequenced for the first time. The total mitogenome length of *E. nipponicus* was 16,173 bp including 13 protein-coding genes, 2 rRNA genes, and 22 tRNA genes. The phylogenetic tree confirmed that *E. nipponicus* belonged to the subsection Heterotremata within Brachyura. This is the first record of the complete mitogenome for the subfamily Eumedoninae.

Eumedoninae Dana 1852, a subfamily of the Pilumnidae, includes 35 species in 13 genera worldwide (Davie 2009); this is well-known as an obligate symbiont of echinoderms (Ng 2014). Of the eumedonid crabs, the genus *Echinoecus* Rathbun (1894) consists of three species, *E*. *nipponicus* Miyake 1939, *E*. *pentagonus* (Milne 1879), and *E*. *sculptus* (Ward 1934) (Ng et al. 2008). These species have only been found in sea urchins thus far. In the present study, we determined the complete mitochondrial genome for a crab symbiotic with sea urchins, *E*. *nipponicus*.

The specimen was collected from the sea urchin *Heliocidaris crassispina* (Agassiz 1864) in the subtidal zone (about 15 m depth) of the southeast coast, Taejongdae, Busan, Korea (35°3′36.41′′N, 129°4′28.33′′E), on 23 September 2016. A voucher specimen was deposited at the National Marine Biodiversity Institute of Korea (MABIK CR00241788). The genomic DNA was extracted from its legs via the method by Asahida et al. (1996). As in the method of Cho et al. (2017), the complete mitochondrial genome sequence was amplified by conducting two independent and overlapping PCR runs with forward and reverse primers. The PCR products were purified and directly sequenced using a set of 23 sequencing primers designed for this study.

The complete mitogenome of *E. nipponicus* was 16,173 bp in length (GenBank accession no. MG574831), and it encoded 37 genes (13 protein-coding genes, 2 rRNA genes, and 22 tRNA genes) and 2 non-coding regions of 945 bp. The base composition of the mitogenome was 35.6% for A, 18.5% for C, 10.2% for G, and 35.6% for T. The initiation codons of the genes contain ATG (*cox1–3*, *atp8*, *nad2–6*, *nad4L*, *cob*), ATT (*atp6*), and ATA (*nad1*). The stop codons contain TAG (*atp8*), TAA (*atp6*, *cox3*, *nad2–4*, *nad4L*, *nad6*), TA (*nad1*, *nad5*), except that some genes (*cox1*, *cox2*, *cob*) terminated with T––.

To confirm the phylogenetic position of *E. nipponicus*, 17 representative species were used, which were in the subsection Heterotremata, based on the mitogenome sequences available in GenBank with two taxa of the subsection Thoracotremata as outgroups. A phylogenetic tree was generated by the maximum likelihood and Bayesian methods in RAxML 7.0.4 (Stamatakis 2006) and MrBayes 3.1.2 (Ronquist and Huelsenbeck 2003) software, with the nucleotide sequence matrix obtained from 11 concatenated protein-coding genes, after excluding ambiguously alignable genes. The tree clearly supports phylogenetic relationships of the subsection Heterotremata within the Brachyura. Moreover, it has shown that *E. nipponicus* is a member of the Heterotremata, and is clustered together with species of the family Bythograeidae (Figure 1).

**Figure 1.**
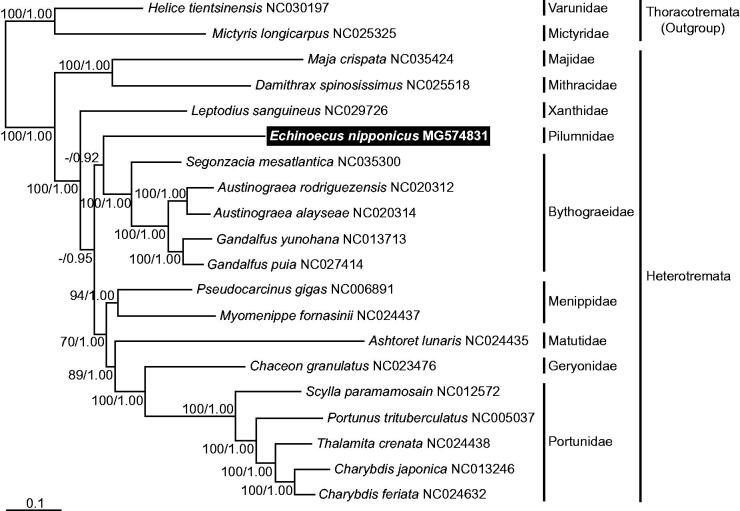
A maximum-likelihood (ML) tree was inferred from the mitogenomic sequences of the representative species belonging to the subsection Heterotremata, with two taxa in the subsection Thoracotremata as outgroups. Phylogenetic analyses were constructed by the sequence matrix based on unambiguously aligning regions of the first and second codon positions of the 11 protein-coding genes. Bootstrap values above 50% in the ML analysis and posterior probabilities above 0.90 in the Bayesian inference (BI) analysis were shown at each node. The symbiotic crab *E. nipponicus*, which was examined in this study, is highlighted in black.

This is the first record of the complete mitogenome for a symbiotic crab *E. nipponicus*, belong to the subfamily Eumedoninae within the Pilumnidae, and it provides useful genetic information for studies not only related to coevolution but also specific interactions between the host sea urchins and symbiont crabs.
